# Added value recyclability of glass fiber waste as photo-oxidation catalyst for toxic cytostatic micropollutants

**DOI:** 10.1038/s41598-019-56836-7

**Published:** 2020-01-10

**Authors:** Gheorghe Nechifor, Eugenia Eftimie Totu, Aurelia Cristina Nechifor, Lucian Constantin, Alina Mirela Constantin, Mihaela Elena Cărăuşu, Ibrahim Isildak

**Affiliations:** 1 Faculty of Applied Chemistry and Material Science, Polytechnic University of Bucharest, 060042 Bucharest, Romania; 2 National Research and Development Institute for Industrial Ecology – ECOIND Bucharest, 71-73 Drumul Podul Dambovitei Str., 060652 Bucharest, Romania; 3 Department of Public Health and Management, Faculty of Dental Medicine, Grigore T. Popa University of Medicine and Pharmacy, 700115 Iasi, Romania; 4 Department of Bioengineering, Faculty of Chemical and Metallurgical Engineering, Yildiz Technical University, 34210 Esenler-Istanbul, Turkey

**Keywords:** Pollution remediation, Environmental monitoring

## Abstract

There is an increased interest in recycling valuable waste materials for usage in procedures with high added values. Silica microparticles are involved in the processes of catalysis, separation, immobilization of complexants, biologically active compounds, and different nanospecies, responding to restrictive requirements for selectivity of various chemical and biochemical processes. This paper presents the surface modification of accessible and dimensionally controlled recycled silica microfiber with titanium dioxide. Strong base species in organic solvents: methoxide, ethoxide, propoxide, and potassium butoxide in corresponding alcohol, activated the glass microfibres with 12*–*13 µm diameter. In the photo-oxidation process of a toxic micro-pollutant, cyclophosphamide, the new composite material successfully proved photocatalytic effectiveness. The present work fulfills simultaneously two specific objectives related to the efforts directed towards a sustainable environment and circular economy: recycling of optical glass microfibers resulted as waste from the industry, and their usage for the photo-oxidation of highly toxic emerging micro-pollutants.

## Introduction

Inorganic-inorganic oxide-type composite materials offer the most interesting technical solutions in many physicochemical processes of bio-degradation, coating, separation, or catalysis^1,2^. A particular case is presented by the silicon dioxide-titanium dioxide (TiO_2_-SiO_2_) composites that combine the distinctive characteristics of the two oxides. Thus, silicon dioxide is a technically-economically accessible material and can be made in the form of spherical particles^3^, nanofibers^4^, nanotubes^5^, or films^6^, thus being used as an active material, adsorbent or support for other materials with higher selectivity^7^. Titanium dioxide prepared in predetermined forms: spheres^8^, nanotubes^9^, nano-threads^10^, or films^11^ could be used as an adsorbent or covering, but especially like catalytic material^12^. Of course, the combination of the two oxides’ properties has been used for the most advanced applications: photocatalytic degradation of organic/pharmaceutical pollutants^13^, fuel cells^14^, membrane reactors^15^, bioreactors^16^, advanced separations^17^ and clinical devices^18^.

**Figure 1:**
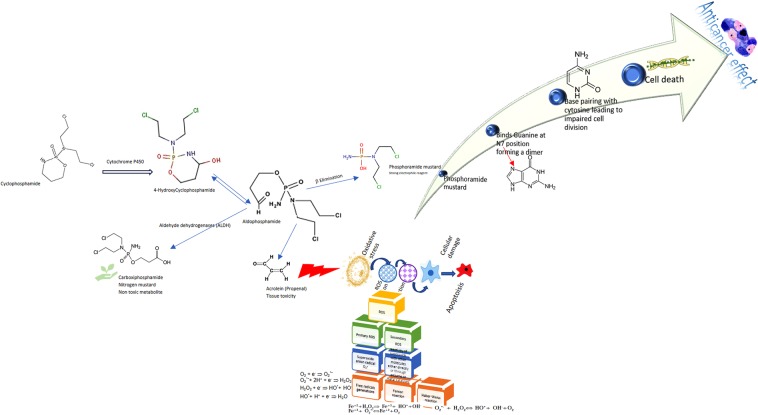
Schematic action mechanism of cyclophosphamide.

Environmental researches proved that the widely used cytostatic drug, N, N-bis(2-chloroethyl)-1,3,2-oxazaphosphinan-2-amine 2-oxide known as cyclophosphamide, which is a cyclic amide, produces residues with mutagenic action. Although mainly known as an anticancer drug, cyclophosphamide is also administrated frequently as an anti-inflammatory or immunolytic agent for rheumatoid arthritis^19^, or nephrotic syndrome^20^, systemic lupus erythematosus^21^, or systemic sclerosis lung fibrosis^22^. This important cytostatic compound administrated for autoimmune diseases, and cancer chemotherapy, as well as its metabolites, are released into effluents that reach surface and ground waters^23^. The cyclophosphamide action through nucleophilic compounds’ alkylation and metabolic activation result in dramatic side-effects like carcinogenic behavior and genotoxic effect^24^. Figure 1 presents the specific action and the activation mechanism of cyclophosphamide.

The action mechanism of cyclophosphamide, according to Figure 1, can provoke the DNA damage. The alkylating drug bio-action is triggered by P450 cytochrome when results 4-hydroxyl cyclophosphamide. Following a ring-opening reaction, the metabolically activated cyclophosphamide will further generate the aldophosphamide, which forms the phosphoramide mustard (active metabolite) and acrolein^25^. The cyclophosphamide’s metabolite, phosphoramide mustard, presents anticancer activity despite its toxicity. The cytotoxic activity is due to the protein and DNA alkylation generated by the cyclophosphamide reactive metabolites^26^. The activated metabolites, as shown in Figure 1, act on both healthy and cancerous cells.

In Figure 1, there is signaled the formation of reactive oxygen species (ROS). The action of either some cytochrome or mitochondria – mitochondrial pathway could explain the ROS presence. When the oxidative forces imbalanced the antioxidant system action of the body, oxidative stress occurs. Such a detrimental body state is due to the presence of free radicals generated by the reactive oxygen metabolites. *In vivo*, oxygen molecules are reduced, forming highly reactive oxygen species as hydrogen peroxide, superoxide radical, or alkoxyl radicals, among others. In the case of excess production of such highly reactive and unstable radicals, the body antioxidant defense mechanism is overwhelmed by the reactive oxygen species presence and the oxidative stress occurs^27^. The reactive oxygen species’ accumulation could initiate cell apoptosis. As a consequence, the usage of cyclophosphamide was proved to generate oxidative stress, finally inducing cell apoptosis^26^.

Therefore, being a hazardous drug, cyclophosphamide has to be removed from any water, as it considered to be a toxic micro-pollutant due to its adverse effects on human health through the drinking water^28^ and wastewaters^29^. This micro-pollutant is mainly present in wastewaters due to the resulting industrial used waters from the organic synthesis of the cytostatic drugs where could be identified the cyclophosphamide’s specific active principles. The patients treated with cyclophosphamide release through the biological fluids (urine) either the drug metabolites, either a part of the untransformed cytostatic drug. Therefore, a second important pollution source is represented by the discharged wastewaters from the oncological treatment clinics. The currently applied treatments of the industrial and clinical resulting wastewaters suppose the application of different physical and chemical processes, such as adsorption, chemical and UV oxidation^30^, membrane separations, flocculation, biodegradation^31^, which are costly and do not provide a specific efficiency for reducing the cyclophosphamide concentration to the legally imposed levels – less than 1 µg/dm^3^.

The conventional method applied, the ozonation, inactivates the cyclophosphamide quite slowly, its destruction taking place in time. In consequence, such cytostatic drug and its metabolites could remain in surface or ground waters, and from there, they could be found in drinking water. Experimental data showed that about 10 g cyclophosphamide intake during 3–6 months rise by 1.5 factor the environmental risk, while the threshold level based on a risk assessment data for active carcinogenic compounds is 10 ng/L^32^. Selective determinations highlighted that through drinking water, there is a possible intake lifetime risk of 10^–5^ or less than 10^−6^ ^33^. However, as mentioned above, not always the cyclophosphamide could be efficiently biodegraded. In such a context, it is of utmost importance to develop affordable technologies for limiting and reducing the presence of cyclophosphamide into wastewaters^34^.

Although the synthesis of silicon dioxide in various geometric forms has been extensively studied and available technical solutions have been found at industrial scale^35^, some economic aspects determined in particular by the high energy consumption and the environmental protection remain a significant challenge. The specific case of using silicon dioxide as support in titanium dioxide composites raises the problem of obtaining the silicon in the predetermined form under economically favorable conditions and, if possible, also protecting the environment. For reproducible silica geometrically shaped, we could consider as reliable and abundant source the optical glass fibers manufacturing industry. The European, US, and Asia-Pacific markets for glass fiber increased tremendously during the last years. For instance, the forecast for 2023 of the specific European market is 2.1 billion USD, with an annual growth rate increasing by 2.8% compared with the 2018 glass fiber production^36^. Also, the actual trend of the global glass fiber’s market points toward an annual growth rate exceeding 4% for the period 2019 to 2024^37^. Such significant development of the glass fiber industry raises environmental concerns related to the glass fibers’ waste disposal. Recycling supposes to transform the glass fiber waste into a new product suitable for returning into the economic mainstream and being financially viable in order to implement the proposed procedure. For instance, the environmental legislation, as is the European Union Directive 2008/98/EC set high targets for recycling techniques^29^. The efforts to develop new recycling methods are welcome, much more if such an applied process would allow environmental applications instead of the environmental burden represented by the glass fiber waste. Recognizing the global production level of 325 million fiber kilometers for 2018^36^, the level of waste resulting from cutting the fibers to a preset size became important.

Seeking for a clean environment, the balance between the environment’s protection and manufacturing industry, became a strategic aim. Thus, our research circumscribes to sustained efforts to find viable solutions for ecosystem protection.

In this paper, we propose to re-use silica microfibers (quartz) – a valuable waste from the optical glass fibers industrial facilities, as support material for titanium dioxide to obtain a composite material through the sol-gel method: silica microfibers with adherent titanium dioxide deposits. The efficiency of new composite material has been tested in the photo-oxidation process of cyclophosphamide in the effort to develop an appropriate method for degrading the cyclic amides with carcinogenic, genotoxic, or mutagenic potential. A new heterogeneous (SiO_2_ – TiO_2_ – UV irradiation) treatment solution is welcome, taking into account the imposed limit under 1 µg/L in discharged wastewaters from the drug synthesis industries or cancer treatment clinics for such anticancer drugs.

## Results

### **Complex characterization of quartz fibers decorated with TiO**_**2**_

#### Morphological and structural analysis of SiO_2_ microfibers decorated with nanoTiO_2_ particles

The quartz microfibers used in this study represent a technical-economically accessible source being a by-product of the silica fiber industry whose use for obtaining advanced materials dedicated to various physicochemical processes: selective separations, controlled adsorption, combustion cells or photo-oxidation reactors would be a niche of valorization. The studied silica microfibrils have a morphology (as shown in Supplementary Material – Figure S.1.) constant and ideal for use as a support for active or selective materials as adsorbents or catalysts. The scanning electron microscopy put in evidence the smooth surface of microfibrils with 12–13 μm diameter and 100 μm length. Also, the EDX analysis presented in Supplementary Material – Figure S.2, evidenced for the silica microfibrils a controlled composition.

The grafting of titanium dioxide microparticles on silica microfibers was carried out by *in situ* titanium dioxide generation through the sol-gel method in alcohol and using the appropriate titanates: tetramethoxy-, tetraethoxy-, tetrapropoxy- and tetra-butylated titanate.

**Figure 2:**
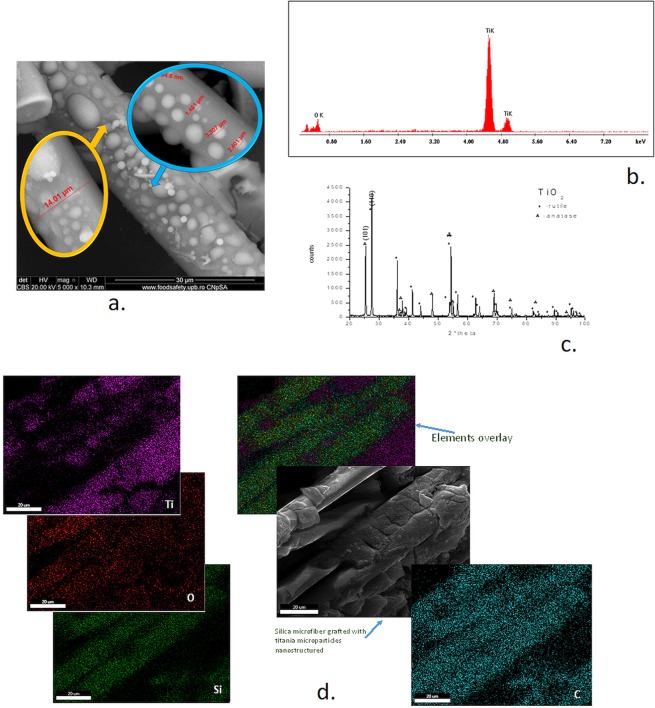
Structural and morphological characteristics of silica microfibrils grafted with titanium dioxide. (**a**) SEM image for dimensional analysis of the titanium dioxide grafted on silica microfibers; (**b**) EDX analysis of the titanium dioxide particles grafted on the silica microfibres; (**c**) XRD analysis of TiO_2_ nanoparticles covering the silica microfibers; (**d**) Elemental mapping for the silica microfiber decorated with TiO_2_.

In Figure 2 are presented the morphological features of the quartz microfibers decorated with titanium dioxide resulting from the tetra-butyl-titanate - tert-butyl alcohol couple. The grafted titania presented dimensions of 1–3.5 μm, while the microfibres diameters are slowly increasing towards 14 μm (Figure 2a).

The EDX spectrum, Figure 2b, highlights the composition of the nanostructured microparticles deposited onto silica microfibers. The diagram puts in evidence the presence of titanium dioxide.

Figure 2c displays the XRD pattern of the titanium dioxide that decorates the silica microfibrils. The X-rays diffractogram highlights the presence of a mixed-phase for nanotitania, being recorded the characteristic peaks for two structural forms of TiO_2_, namely: anatase and rutile. Applying the calculation relationship (Eq. 3) for particle dimension, it resulted in the average values for anatase (24 nm) and rutile (29 nm) crystallites.

The elemental mapping, Figure 2d, for the silica microfiber decorated with TiO_2_ evidencied the presence of Ti, Si, and O, as well as the C element. The recorded presence of carbon could be explained by the retention within the material pores and interstices traces of the organic material used during the synthesis stage, although the thorough washing of the obtained material was appropriately performed.

The percentual content in anatase and rutile phases of the titania crystalline nanoparticles was estimated from the XRD pattern (Figure 2c) by the help of the Eqs. (4) and (5). The calculated values indicated the content of 30.8% anatase and 68.9% rutile for the mixed-phase.

**Figure 3:**
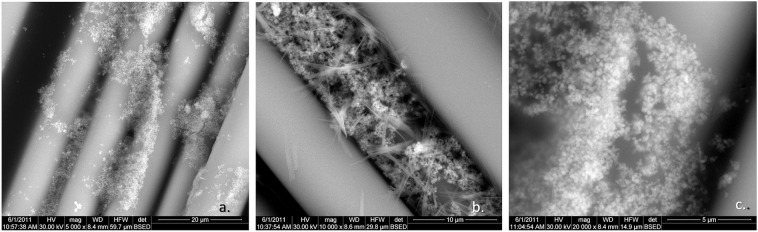
SEM images of non-adherent titanium dioxide nanoparticles resulted from the system: (**a**) titanium (IV) methoxide - methyl alcohol; (**b**) titanium (IV) ethoxide - ethyl alcohol; (**c**) titanium (IV) propoxide - propyl alcohol.

The other tetraalcoxy titanate – alcohol systems considered, lead to the formation of titanium oxide as non-adherent nanoparticles (Figure 3a–c).

#### *Thermal analysis* of SiO_2_ microfibers decorated with nanoTiO_2_ particles

Thermal analysis (TGA and DSC) could provide helpful information regarding the behavior of the SiO_2_ – TiO_2_ system and its stability – Supplementary Material Figure S.3. The performed thermal analysis of the quartz fibers decorated with microparticles nanostructured of TiO_2_ (Figure S.3b) allowed the comparison of the specific characteristics with those of the silica support fibers (Figure S.3a).

The sample of silica microfiber (Figure S.3a) proved to be stable over time, according to the assumption that it does not undergo any transformation. The specimens of silica microfibers decorated with titanium dioxide lost their mass during three stages of heating (Figure S.3b).

#### *UV-Vis –Differential reflectance spectroscopy (DRS*) of SiO_2_ microfibers decorated with nanoTiO_2_ particles

**Figure 4:**
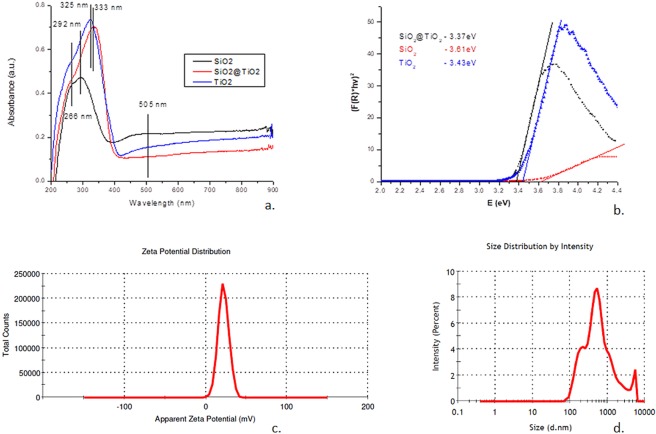
(**a**) DRS spectra for quartz fibers decorated with TiO_2_, quartz microfibers, and TiO_2_ (P25, Degussa). (**b**) Absorbance spectra for the direct electronic transition, F(R)^2^ *vs*. E (eV) of the quartz fibers decorated with TiO_2_, silica microfiber, nanotitania Degussa P25; (**c**) Zeta potential distribution for quartz fibers decorated with TiO_2_; (**d**) Particle size distribution for quartz fibers decorated with TiO_2_.

The DRS spectra for the TiO_2_ grafted on silica microfiber, nanotitania Degussa P25 (as reference), and initial silica microfiber are presented in Figure 4a.

According to the spectra from Figure 4a the cut-off values obtained were 408 nm for quartz fibers decorated with TiO_2_, and 385 nm for the silica microfiber. When the system absorbs radiation with an energy exceeding the band gap of the material, the transfer of electrons from the valence band into the conduction band becomes possible. In Figure 4b it is presented the variation of ${(F(R)\cdot h\upsilon )}^{2}$ versus *E(eV)* for the direct electronic transition. For studied samples: quartz fibers decorated with TiO_2_, silica microfiber, and nanotitania Degussa P25, the band gaps were obtained by extrapolating the linear section to the E axis. The silica microfibers grafted with nanotitania activated with 408 nm radiation showed a band gap of 3.37 eV. This value is lower compared with the band gap values recorded for silica microfiber, 3.61 eV, and TiO_2_ Degussa P25, 3.43 eV. The latest band gap values are higher than the value characterizing the obtained material. Therefore, we could consider that the obtained quartz fibers decorated with TiO_2_ present a higher degree of crystallinity, and, as a consequence, an improved photocatalytic activity is expected.

#### *Electrokinetic characteristics of SiO*_2_*microfibers decorated with nanoTiO*_2_*particles*

When used a certain material in suspension, due to the random movement of the particles, it could aggregate if the repulsive forces are not sufficient. The repulsive forces between particles increase as zeta potential increases. In consequence, zeta potentials allow assessing the stability of a given system. The distribution of potential zeta for the quartz fibers decorated with TiO_2_ is presented in Figure 4c. The diagram shows that the obtained material has a zeta potential of 22.0 mV. In Supplementary Material, Figure S.4 presents the variation of zeta potential with the pH change for quartz fibers decorated with TiO_2_. The iso-electric point (IEP) for the studied material calculated from the recorded experimental data was IEP_SiO2-TiO2_ = 2.6. Also, the zeta potential is particle-size dependent. Figure 4d presents the particle size distribution. The size distribution by volume is introduced in Supplementary Material Figure S.5. According to literature, the photocatalytic activity of nano-TiO_2_ depends on the particle size for the same particle surface area^38^. Therefore, it is expected a similar dependence for the silica microfiber – nano-TiO_2_ composite. The average diameter was ranging between 185.5 nm to 4836 nm underlying the specific deposition of nanotitania onto silica when the nanoparticles agglomerates, forming a nanostructured decoration.

#### *Brunauer-Emmett-Teller (BET) analysis of SiO*_2_*microfibers decorated with nanoTiO*_2_*particles*

**Figure 5:**
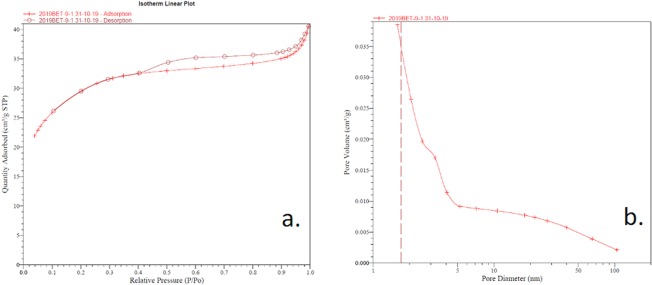
Absorption-desorption plot (**a**) and pore size and volume distribution (**b**) of quartz microfiber decorated with nanotitania.

Figure 5 depicts the N_2_ adsorption/desorption type IV isotherm and pores size distribution. In Figure 5a, the isotherm describing the material behavior presents a hysteresis loop.

The determined surface area was 101.62 ± 1.38 m²/g, while the pores volume was 0.036 cm³/g. Figure 5b displays the pore size distribution of the obtained material. From the distribution curve, it was determined the average dimension of pores – 2.90 nm.

#### *Thesurfacestructureofsilicamicrofibersdecoratedwithnano-TiO*_*2*_

**Figure 6:**
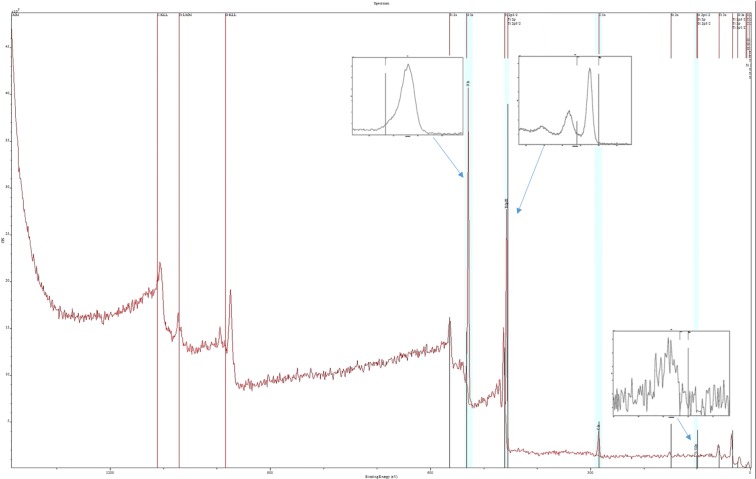
XPS spectra for the silica microfibers grafted with nanotitania.

The XPS probed the chemical composition of the grafted silica microfibers, Figure 6. The surface analysis of the SiO_2_ microfiber grafted with nano-TiO_2_ could be explained by the balance between the relatively hydrophobic surface of TiO_2_ and the hydrophilic nature of the conditioned SiO_2_ surface when OH^−^ amount increased^39^. The presence of the Ti peak on XPS spectra sustains the grafting of TiO_2_ nanoparticles on the silica microfibers surface. It was observed that the specific Si peaks became weaker, proving the covering of the microfiber. The carbon C1s presence was due to the synthesis pathway.

The surface of silica microfiber was hydrolyzed, thus being covered with hydroxyl groups, and favoring the titania nanoparticles grafting. The Si atoms are involved in the formation of Si-O-Ti bond, and due to the preferential transfer of electrons from Ti to O, it resulted in larger electron densities for Si. As a consequence, the grafting of the titania nanoparticles leads to a decrease of Si2p binding energy. Much more, at the interface, there is an indication that a bond between Ti and Si was formed, rather than physical adsorption.

#### *Determinationofthecatalyticcharacteristicsof silicamicrofibersdecoratedwithTiO*_*2*_

In order to determine the photocatalytic properties of the silica microfibers grafted with TiO_2_, there was followed up the effect of significant factors on the photodegradation of the cytostatic drug. Key parameters in photocatalysis that were investigated in this study are the catalyst quantity loaded, initial concentration of the analyte, and the influence of irradiation time.

*Effect* *of* *catalyst* *amount*. For establishing the appropriate catalyst amount, herein quartz fibers decorated with titanium dioxide, it has been followed up the degradation rate for CP versus the catalyst dose for 30 min irradiation (UV) and considering a CP concentration of 7.25 × 10^−5^ mol/L. The variation of CP’s degradation rate with the increase of the catalyst amount – silica decorated microfibers, is presented in Supplementary Material– Figure S.7.

**Figure 7:**
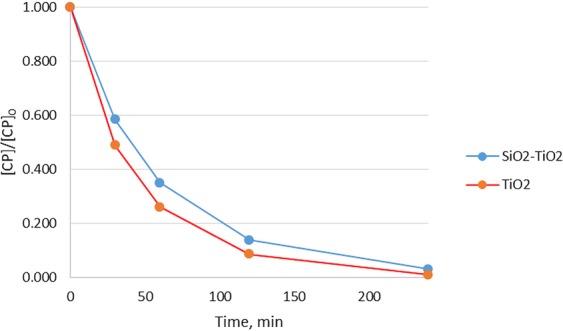
Normalized CP residual concentration vs. irradiation time.

*Effect* *of* *irradiation* *time*. The effect of irradiation time was studied following the photocatalytic behavior of [SiO_2_-TiO_2_] and TiO_2_ Degussa P25 over a 30–240 min. range. The dose of the catalyst in both cases was 100 mg/L. Figure 7 introduces the obtained experimental results related to the variation of normalized CP residual concentration with irradiation time.

*Effect* *of* *initial* *CP* *concentration*. Table 1 contains the data related to the influence of CP’s initial concentration upon the photodegradation efficiency. Irradiation time was set to 30 minutes, while the dose of the catalyst [SiO_2_-TiO_2_] was 100 mg/L.

**Table 1:** Initial CP concentration vs. degradation efficiency for silica microfibers – titania decorated catalyst; dose 100 mg/L and irradiation time of 30 min.

[CP]_0_		[CP]		η_CP_
mg/L	mol/L	mg/L	mol/L	%
1.04	3.98 × 10^−6^	0.46	1.76 × 10^−6^	55.77
2.12	8.12 × 10^−6^	0.96	3.68 × 10^−6^	54.72
5.04	1.91 × 10^−5^	2.29	8.77 × 10^−6^	54.56
9.83	3.77 × 10^−5^	5.23	1.88 × 10^−5^	49.95
18.94	7.25 × 10^−5^	11.08	4.25 × 10^−5^	41.50
52.13	2.00 × 10^−4^	30.93	1.19 × 10^−4^	40.67

Applying the specific reaction rate equation, the obtained data allowed to model the photochemical system behavior through Langmuir – Hinshelwood kinetic.

## Discussions

### *Morphological and structural analysis of SiO*_2_*microfibers decorated with nanoTiO*_2_*particles*

The SEM analysis performed on the silica microfibrils put in evidence a uniform and constant surface aspects (Supplementary – Figure S.1) as well as a smooth surface characterized by 12–13 μm for diameter and 100 μm for length (Supplementary – Figure S.1.c).

For the titanium dioxide microparticles grafting on silica microfibers (Supplementary – Figure S.2.a.), the latest was superficially activated with potassium alkoxides in the corresponding alcohols (methanol, ethanol, propanol, and t-butanol.) The surface’s activation creates primary centers on the microfibrils subsequently introduced into the titanium dioxide grafting medium (Supplementary - Figure 2.b).

For grafting the TiO_2_ onto the silica microfibers’ surface, as presented in Section “*Procedure for preparing quartz fibers decorated with TiO*_2_^”^, there were four systems considered: tetramethoxy-titanate/methyl alcohol, tetraethoxy-titanate/ethyl alcohol, tetrapropoxy titanate propyl alcohol, and tert-butylate titanate/tert-butyl alcohol. The tert-butylated-titanate/ tert-butyl-alcohol couple was the only system that resulted in adherent grafting and relatively well-distributed titanium dioxide onto the surface of quartz microfibers (Figure 2). As shown in Figure 2a, the quartz microfibres dimension increased towards 14 μm due to the formation of titania nanostructured microparticles of 1–3.5 μm over the smooth silica surface.

The EDX analysis performed on the decorated silica microfibers (Figure 2b) confirmed the formation of titania onto the quartz surface. The XRD analysis showed that from the structural point of view, the titania decoration covering the silica microfibres consists of two forms: anatase and rutile (Figure 2c). The titania nanoparticles are presenting the calculated dimensions of 29 nm (anatase phase) and 33 nm (rutile phase) agglomerate to form microparticles, as evidenced by SEM images (Figure 2a).

Based on the specific properties of each structural phase of TiO_2_, there are particular applications like UV filters and photocatalyst for anatase form or materials with magnetic properties and catalyst for rutile form. It is expected that the obtained SiO_2_ – nanoTiO_2_ hybrid material to potentially act as active photocatalyst. The XRD analysis results show the presence of both phases: anatase and rutile wrapping the silica microfibres. Therefore, it was necessary to calculate the ratio between the two structural forms. The higher content of the rutile phase, 68.9%, provided higher stability to the composite system obtained. The presence of both TiO_2_ phases could enhance photocatalytic activity. It is known the better photocatalytic behavior of anatase variety. However, the rutile nanoparticles added to anatase nanoparticles could improve the hydroxyl groups’ discharge and the electron transfer^40^ and in consequence, the photocatalytic activity.

The other three systems considered for synthesis, tetra-alcoxytitanat – alcohol (see Section 4.3.), lead to the formation of non-adherent titanium dioxide nanoparticles (Figure 3a–c).

### *Thermalanalysis*

The complete thermal analysis performed on the SiO_2_ microfibers – with and without titania decoration, evidenced their stability (Supplementary Material, Figure S.3.a). The residual mass of 99.25% indicates that, eventually, water traces or -OH groups from the surface are lost (Figure S.3.a). The SiO_2_-TiO_2_ sample showed mass losses over three intervals (Figure S.3.b). The first mass loss of 2.98% occurred from the ambient temperature to 175 °C can be attributed to the removal of water molecules (or another volatile solvent) and weakly –OH linked groups to the particle’s surface^41,42^.

In the 175–340 °C range, the oxidative degradation occurred, and the sample lost 3.77% of its weight. The exothermic effect produced during the thermal process had a maximum decomposition rate at 280.2 °C and an area of 232.5 J/g. Most likely, the burning of some organic precursor residues used in synthesis caused this thermal degradation (Figure S.3.b).

The last slow weight loss (0.95% in the range 340–480 °C and a 1.13% residue to 900 °C) occurs due to the thermolysis of the amorphous carbon traces remaining from the oxidative degradation. Also, a slight exothermic effect from 340 to 465 °C, with a maximum at 415.3 °C, and an area of 34.61 J/g was recorded. This is caused both by the slow oxidation of carbon and the transformation of the anatase into rutile phase^43^. The last phase change is presented in the literature ranging between 350 and 1175 °C depending on the synthesis method, the nanoparticle size, or the presence of impurities^43^.

### *Electrokineticstudies*

Modifications of zeta potential are generated by adsorption, ionization, or exchanges of chemical species. Thus, when zeta potential (ξ) decreases (its module), then the system is characterized through a high ionic strength, signifying that a significant number of charge carriers are present. In general, when pH decreases, the surface charge becomes more positive, while the pH increase dictates a more negative charge. It is known that when immersing oxides in aqueous solution, the hydroxyl groups of the metallic cations are not altered if the solution has the pH value at the isoelectric point (IEP) of respective oxide. In our case, the IEP for TiO_2_ was 4.2 mV, and for silica, microfiber is 2.4 mV. As shown in Supplementary Material Figure S.4. for the obtained quartz fibers decorated with nanoTiO_2,_ the value of IEP is 2.6 mV. The experimental work was performed at a pH value higher than IEP, in a basic media for synthesis, and towards neutral during the photocatalytic processes.

In consequence, the hydroxyl group on the surface will dissociate, imparting a negative charge to the surface of the quartz microfibers decorated with TiO_2_ due to the formation of TiO^−^ and SiO^−^ species. The nature of the surface hydroxyl from the silica microfibers decorated with the TiO_2_ surface is an essential factor in the photocatalytic process. However, the IEP value is low compared with the values reported in the literature^38^. As our material has an IEP lower than 7, it has an acidic character. A higher value for zeta potential could secure a stable suspension, while a low value (<10 mV) leads to particle association and instability. For the silica microfibers decorated with TiO_2,_ the value of zeta potential was 22 mV. As a high value for ξ assure a maximum dissociation of the functional groups located on surface and particle dispersion, it is expected for the synthesized composite to have a well-dispersed suspension and the surface chemical species will be readily dissociated.

### *BET analysis of SiO*_2_*microfibers decorated with nanoTiO*_2_*particles*

The BET analysis resulted in a type IV isotherm, Figure 5a, that is specific for porous adsorbents with pores in the area of mesopores and macropores, covering the range of 1.5–100 nm. According to the shape of the hysteresis loop, the system exhibits an H4 type isotherm, when the two branches of the loop are almost parallel and horizontal over an extended range of relative pressure, and the geometry of the pore is similar to a narrow slit-shaped structure.

The mesostructured pores appeared from the nanotitania deposition onto the quartz microfibers when voids were formed. In general, an improved photocatalytic activity is conditioned by the high surface area (101.62 ± 1.38 m²/g) and pore volume.

### *UV*-*Vis*-*DRSanalysis*

The results of DRS analysis for silica microfibers grafted with nanotitania highlighted a band gap of 3.37 eV. Such band gap values could be associated with the crystalline characteristics of the material^44,45^ that are in good agreement with the X-Ray diffractogram, Figure 2c. When grafting the TiO_2_ nanoparticles onto the silica microfibers, for the chemical process:

I $${\rm{Ti}}{({\rm{OR}})}_{4}+{{\rm{4H}}}_{2}{\rm{O}}\rightleftarrows {\rm{Ti}}{({\rm{OH}})}_{4}+{\rm{4R}}({\rm{OH}})$$

an excess of the aqueous solution was used, exceeding the stoichiometric ratio. This situation leads to a nucleophilic reaction between the OH^−^ (from water molecules) and OR^−^ (from alkoxide molecules), thus lowering the possibility to have non hydrolyzed OR^−^ and in consequence, the crystallization of titania is favored.

The titanium oxide variety, rutile, allows a straightforward promotion of electrons from the valance band to the rutile conduction band when exposed to UV excitation, but rapid electron-hole pair recombination accompanies the process. Following the combined processes, there are limited charge carriers that could be involved in various photoreactions occurring at the surface level of the oxide. On the other hand, anatase variety that behaves like an indirect band gap semiconductor allows the generation of more holes and electrons available for photoreactions. In consequence, rutile is not very efficient as a catalyst compared to anatase that presents a substantial amount of electrons and holes reaching its surface. However, there should be specified that such consideration does not take into account any influence of the surface area.

The titania immobilized onto the silica microfibers obtained during this work, present both TiO_2_ varieties forming a type II heterojunction photocatalyst^40^. Therefore, specific anatase-rutile heterojunctions are present when the rapid mass transfer occurs, and the separation efficacy of hole-electron is satisfactory. Based on the specific values of the conduction and valence band potentials, the obtained composition of the grafted titanium oxide allows a two-way charge displacement between anatase and rutile: the generated holes migrate between the valence bands from anatase towards rutile, and the photo-excited electrons move between the conduction bands from rutile into anatase. These charge migrations generate an increase of the electrons and holes that could improve the overall behavior as photocatalyst^12^. As discussed previously, the titanium oxide photocatalyst proved to be efficient in the treatment of wastewater pollutants. The extended applicability of nano-TiO_2_ as photocatalyst is limited due to the rapid recombination of electron-hole pair and a large band gap^44^. In the context of the efforts to overcome these drawbacks, the obtained quartz fibers decorated with TiO_2_ presents a lower band gap, thus enhancing the photocatalytic activity. In the obtained material, the electrons flux occurs from the conditioned SiO_2_ microfiber characterized by a higher band gap (3.61 eV) towards the intrinsically n-type semiconductor TiO_2_ that has a lower value of band gap (3.43 eV).

### *XPSstudies*

The electron density of a chemical species influences the value of its binding energy. A decrease in binding energy implies a higher electron density. As presented in Figure 6, the Ti 2p biding energy for SiO_2_ microfiber-covered with nano-TiO_2_ decreased to 458.0 eV compared with the literature data^46^. The binding energy indicates a change in the chemical environment and an increase of electron densities at the Ti level. In literature has been reported an increase of the Ti2p binding energy for silica microfibers grafted with TiO_2_^46^. The formed Ti-O-Si bond involves the electrons transfer from Ti and Si towards oxygen, and taking into account that Si supplies fewer electrons than Ti, the electron densities level will decrease due to its electrons transfer towards oxygen^46^. The different behavior of our SiO_2_-TiO_2_ system is due to the distinct synthesis method applied. We could consider that we have predominant Ti-O-Ti bonds. Taking into account that the XPS analysis can provide information about the surface top layers (10 nm depth), and we do not have a thin film deposition of nanotitania, but microparticles nanostructured as resulted from scanning electron microscopy, it means that the detected presence of Si was possible due to incomplete coverage of the silica microfiber with titania nanoparticles. Therefore, to a certain extent, it could be assumed that in any area of SiO_2_ microfiber - nano TiO_2_ composite surface, only SiO_2_ or TiO_2_ brings its contribution to the XPS spectrum.

The XPS spectra (Figure 6) displays the shift of the Si^4+^ peak towards lower binding energy (102.0 eV) compared to the bulk SiO_2_ (103.3 eV)^47^. This shift of the SiO_2_ surface binding energy could be assigned to the lower binding energy of the Si placed at the interface during the TiO_2_ grafting onto SiO_2_, and to the formation of Ti-O-Si bond replacing Si-O-Si bond. Although the binding energies for Ti2p peaks put in evidence that the titanium is in its Ti^4+^ state, there is a shift towards lower binding energies from the characteristic value for Ti^4+^/ TiO_2_ bulk of 458.5 eV^47^ to 458.0 eV for Ti2p_3/2_, while the peak for Ti2p_1/2_ is placed at 463.4 eV^44,48^. Also, the O1s peak was shifted to lower values, with a maximum at 529.5 eV following an increased negative charge density at O level partially due to a higher degree of covalence of Si-O bond compared to the ionic character of Ti-O bond^48^.

The reduced decrease in the binding energy value for Ti2p_3/2_ sustains the presence of titanium in its Ti^4+^ state^49^. The characteristic Ti2p spin-orbit splitting structure, with a 5.4 eV separation between peaks, was highlighted in Supplementary Material, Figure S6b being in concordance with the data reported in the literature^46^. Such behavior indicates lower binding energy for titanium species compared with the titania bulk. The Ti2p and Si2p bind energies shift towards lower values, indicating higher charge density on Ti and Si following the formation of Ti-O- and Si-O- terminal bonds.

The specific signal for O1s resulted from overlapping the specific bands for both oxides and hydroxyl as well (Supplementary Material, Figure S6a). The values characteristic for the binding energy for O1s in SiO_2_ (533.0 eV) and TiO_2_ (529.9 eV), as well as the contribution of OH^−^ (531.0 eV), are also well shifted towards lower binding energies, respectively to 529.5 eV for TiO_2_O1s and 530.0 eV for OH^−^ O1s. The exhibited O1s peaks could be ascribed to the surface OH (530.0 eV) and the O^2−^ species of titania (529.5 eV). The success of the material as photocatalyst depends on the reactivity, chemical state, and hydroxyl groups.

Also, we should notice the presence of the C1s signal (at 285.0 eV) corresponding to the C-O in alcohol, signifying that the material morphological structure allowed the accommodation of some traces of organic alcohols used during the material synthesis, although thorough wash of the material was performed.

For the surface ratio of O, Ti, and Si calculated as [A_O_/(A_Ti_ + A_Si_)] from XPS data, it was obtained 1.53. It has been reported that for a ratio smaller than 2.00, the surface presents oxygen vacancies^50^. The presence of oxygen vacancies generates defects able to act as a trap for holes^51^. In consequence, the holes and excited electrons recombination is limited, implying an improved charge transfer.

### *Photocatalyticcharacteristicsofsilicamicrofibers* decorated *withTiO*_*2*_

As shown in section dedicated to the “*Effect of catalyst amount,”* it could be followed-up the variation of cyclophosphamide (CP) degradation rate with the catalyst dose for an initial CP concentration of 7.25 × 10^−5^ M, and an irradiation time of 30 min – see Supplementary Material, Figure S.7. The reaction rate increased with the increase of catalyst amount up to [SiO_2_-TiO_2_]  = 100 mg/L. The increase of the catalyst surface area available for photocatalysis could explain the observed behavior.

However, a further increase of catalyst amount led to the decrease of reaction rate due to the light scattering effect with a negative impact on photoexcitation efficiency and, therefore, on the CP degradation rate. This trend was also observed in previous studies on CP photocatalytic degradation^52^ when there was an optimum catalyst amount established at 100 mg/L. The optimum catalyst dose assures a CP degradation of 41.5% after 30 min of irradiation.

The behavior of silica microfibers decorated with TiO_2_ was followed up by comparison with the known catalyst TiO_2_ Degussa P25, when equal catalyst doses have been used, namely 100 mg/L. The experimental results, normalized CP residual concentration with irradiation time, presented in Figure 7, were obtained for 30–240 min irradiation time. It has been proved that prolonged irradiation time has a positive effect on CP degradation efficiency. In the case of SiO_2_-TiO_2_ catalyst, a CP degradation efficiency of 96.99% was obtained after 30 minutes of irradiation for an initial CP concentration of 7.25  × 10^−5^ M. The determined value is satisfactory compared with CP degradation efficiency of 99.04% obtained when the same catalyst dose of TiO_2_ Degussa P25 was used in similar experimental conditions. The fact that TiO_2_ in the SiO_2_-TiO_2_ material is in a much lower amount compared with TiO_2_ Degussa P25 suggests that SiO_2_-TiO_2_ could be a better photocatalyst.

The composite material obtained takes advantage of the silica substrate presenting corrosion protection, excellent mechanical strength, or high thermal stability. In the meantime, the titanium oxide semiconductor can absorb UV radiation to photo-generate electrons, and the corresponding holes that produce hydroxyl radicals when immersed in aqueous solutions. As a consequence, the photocatalytic activity could be improved due to an increased presence of OH•. Taking into account that the OH• could be produced through the chemical reaction between water and a surface highly acidic, it is expected that the obtained composite would absorb significant amounts of hydroxyl radicals compared to the titanium oxide. This behavior is substantiated by the presence of Si-O-Ti bonds that are acidic, thus conferring a high acidic characteristic to the silica microfibers decorated with nanotitania. Therefore, it is expected that the obtained composite material to have improved photochemical activity.

Kinetic curves for CP degradation when using both SiO_2_-TiO_2_ and TiO_2_ Degussa P25 were linearized using a pseudo-first-order kinetic, and the constant rates in both cases were calculated for an initial CP concentration of 7.25 × 10^−5^ M and catalyst dose of 100 mg/L (Supplementary Material – Figure S.8). The constant rates calculated from the slopes of the graphs were 1.97 × 10^−2^ M/min in the case of TiO_2_ Degussa P25 and 1.51 × 10^−2^ M/min in the case of SiO_2_-TiO_2_ photocatalyst.

Further studies followed the influence of CP’s initial concentration upon the degradation efficiency (Table 1) when the [SiO_2_-TiO_2_] concentration was 100 mg/L, and the irradiation time applied was 30 min. In the same working conditions, the increase of initial concentration led to the decrease of the degradation efficiency because higher CP levels increase the content of degradation intermediates, which compete with CP for hydroxyl radicals’ consumption.

The Langmuir – Hinshelwood kinetic model can describe CP degradation using SiO_2_-TiO_2_ catalyst starting from the following reaction rate Eq. (1):

1 $${r}_{0}=\frac{{k}_{r}{K}_{ad}{[CP]}_{0}}{1+{K}_{ad}{[CP]}_{0}}$$

where:

r_0_ = initial CP degradation rate (M min^−1^)

[CP]_0_ = initial CP concentration (M)

k_r_ = rate constant for CP photocatalytic degradation (min^−1^)

K_ad_ = equilibrium constant of CP adsorption–desorption on photocatalyst surface(M^−1^)

The above-mentioned Eq. (1) could be rearranged as follows Eq. (2):

2 $$\frac{1}{{r}_{0}}=\frac{1}{{k}_{r}{K}_{ad}{[CP]}_{0}}+\frac{1}{{k}_{r}}$$

The Eq. (2) allows to plot the relationship: 1/r_0_=f(1/[CP]_0_), as linear dependence, in order to calculate the values for the rate constant k_r_ and adsorption – desorption equilibrium constant K_ad_. From the linear representation, the intercept gives 1/k_r_ while the slope is 1/k_r_K_ad_ (Supplementary Material – Figure S.9).

The adsorption-desorption equilibrium constant K_ad_ was 3698 M^−1,^ and the constant rate k_r_ was 5.10 × 10^−6^ M min^−1^. By correlating the experimental data with the Langmuir – Hinshelwood equation results that the degradation of adsorbed CP occurs onto the nano-TiO_2_ surface where the photogenerated radicals are also adsorbed.

The values of k_r_ and K_ad_ obtained for SiO_2_-TiO_2_ photocatalyst are similar to those obtained in our previous works for CP degradation using TiO_2_ in anatase form^53^, which suggests that the new material could be a promising and efficient catalyst for CP’s degradation.

Generally, when referring to the cyclophosphamide’s photooxidation mechanism, it should take into consideration both intermediate and final degradation products (carbon dioxide, ammonium, phosphate, and chloride anion).

In a previous work of our group, based on identified intermediates/degradation by-products^54^, there was proposed a possible alternative for the CP degradation pathway under UV-Vis/silica microfiber decorated nano-TiO_2_ assisted photocatalysis – Supplementary Material Figure S.10.

The photodegradation pathway supposes a direct action on CP. The following processes Eqs. (II) to (XI) for the photo mechanism could apply^55^:

II $${{\rm{S}}{\rm{i}}{\rm{O}}}_{2}{@{\rm{T}}{\rm{i}}{\rm{O}}}_{2}+{\rm{h}}\nu \to {{\rm{T}}{\rm{i}}{\rm{O}}}_{2}({{\rm{e}}}_{{\rm{C}}{\rm{B}}}^{-})+{{\rm{T}}{\rm{i}}{\rm{O}}}_{2}({{\rm{h}}}_{{\rm{V}}{\rm{B}}}^{+})$$

III $${{\rm{H}}}_{2}{{\rm{O}}}_{{\rm{ads}}}\rightleftarrows {{\rm{H}}}_{{\rm{ads}}}^{+}+{{\rm{HO}}}_{{\rm{ads}}}^{-}$$

IV $${{\rm{h}}}_{{\rm{VB}}}^{+}+{{\rm{H}}}_{{\rm{ads}}}^{+}+{{\rm{HO}}}_{{\rm{ads}}}^{-}\to {{\rm{H}}}^{+}+{{\rm{HO}}}^{\cdot }$$

*Generation of superoxide anion radicals*, O_2_^·−^

V $${({{\rm{O}}}_{2})}_{{\rm{ads}}}+{{\rm{e}}}_{{\rm{CB}}}^{-}\to {{{\rm{O}}}_{2}}^{\cdot -}$$

*Formation of hydroxyl radicals*, HO^·^(VI)

VII $${{{\rm{O}}}_{2}}^{\cdot -}+{{\rm{H}}}_{2}{\rm{O}}\rightleftarrows {{{\rm{HO}}}_{2}}^{\cdot }+{{\rm{HO}}}^{-}$$

VIII $$\begin{array}{c}{{{\rm{HO}}}_{2}}^{\cdot }+{{{\rm{HO}}}_{2}}^{\cdot }\to {{\rm{H}}}_{2}{{\rm{O}}}_{2}+{{\rm{O}}}_{2}\\ {{\rm{H}}}_{2}{{\rm{O}}}_{2}+{{\rm{e}}}^{-}\to {{\rm{HO}}}^{-}+{{\rm{HO}}}^{\cdot }\end{array}$$

*Action paths on CP:HO* ^•^ *radicals oxidative action on the CP*

IX $${{\rm{CP}}+{\rm{HO}}}^{\cdot }\to {{\rm{H}}}_{2}{{\rm{O}}+{\rm{CP}}}^{\cdot }\to degradation\,products$$

*Holes direct oxidative action on CP*

X $${\rm{CP}}+{{\rm{h}}}_{{\rm{VB}}}^{+}\to {{\rm{CP}}}^{\cdot +}(oxidation\,products)$$

*Photodecomposition CP*

XI $${{\rm{CP}}}^{\cdot +}\to CP\,photodegradation\,products$$

**Figure 8:**
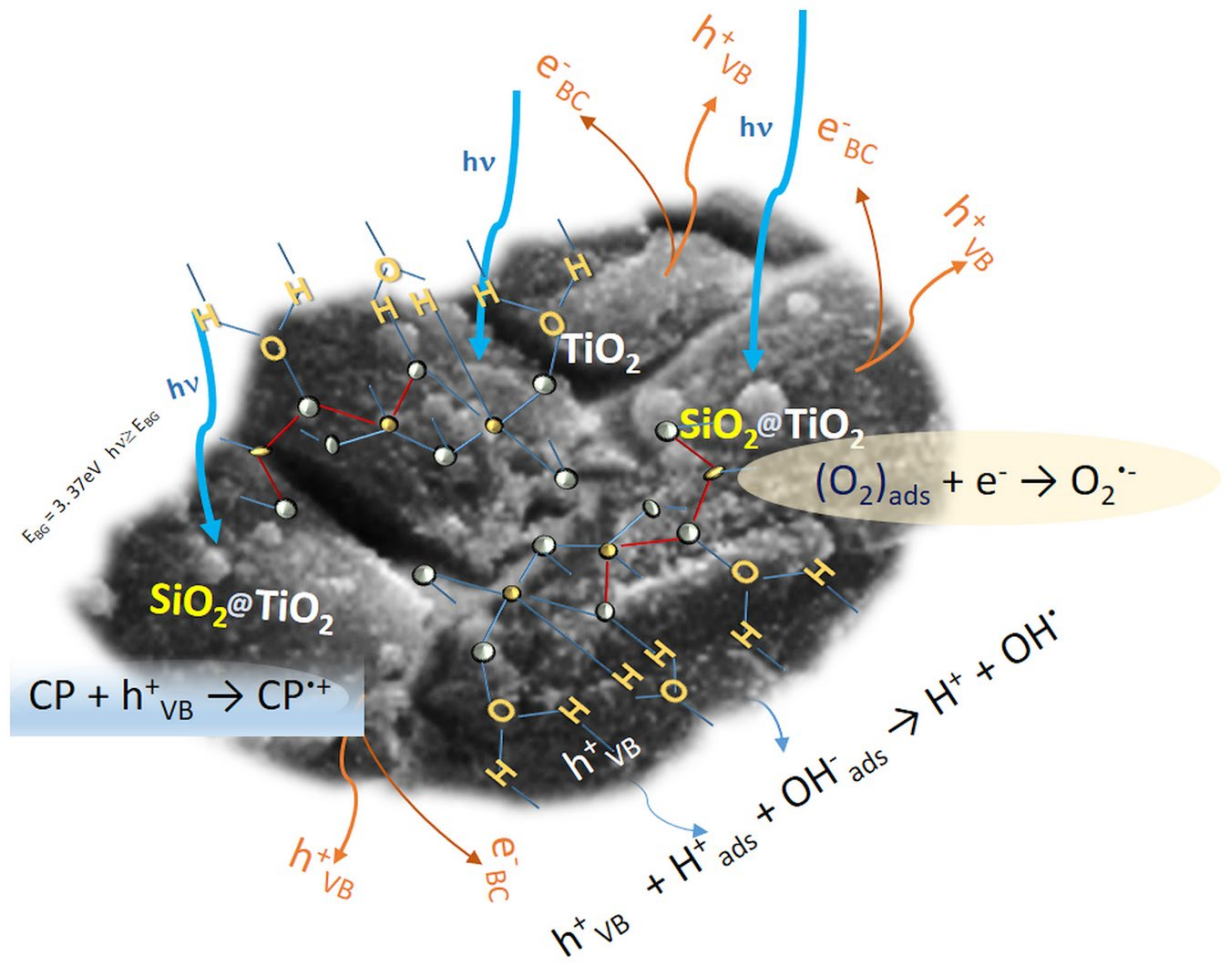
Speciation and chemical states on the surface of SiO_2_ microfibers decorated with nano-TiO_2_.

Based on the surface characteristics and the proposed photochemical reaction sequence, in Figure 8 is presented the speciation at the surface of the silica microfibers decorated with nanotitania.

## Conclusions

The affordability and the relatively constant physical characteristics: dimensions, and shape, recommend the usage of quartz optic fibers for various applications. The microfibers used in this study had a diameter of 12–15 µm and a maximum length of 100 µm. After their activation in alcoholic - potassium alcoholate solution, the microfibers can be adherently decorated with *in situ* generated titanium dioxide applying the sol-gel method using the titanates of their corresponding alcohols. The quartz microfibers decorated with titanium dioxide have been morphologically and structurally characterized (SEM, XPS, UV-DRS, XRD, EDX, ξ-potential, BET, and TGA). Zeta potential allowed the study of material stability. Nanotitania coating of the quartz microfibers leads to IEP = 2.6 mV, resulting in improved electrophoretic mobility. The value of zeta potential, 22 mV, indicates a stable suspension of the coated quartz nanofibers. Due to the nanotitania coating, the specific surface area was 108 m^2^/g. The XPS analysis put in evidence the formation of the Ti-O-Si bond and the existence of hydroxyl groups as well as oxygen vacancies on the composite material surface. The decoration of silica microfibers with nanotitania lowered the band gap to 3.37 eV, thus presenting improved characteristics for photochemical reactions.

The quartz microfibers decorated with titanium dioxide have been preliminarily tested in the photo-oxidation process of cyclophosphamide with promising results regarding the efficiency and affordability of the new catalyst composite.

Due to the thin limit between the toxic effect and the targeted efficacy of the cyclophosphamide, and the recorded presence in wastewaters of its toxic metabolites, essential efforts are oriented towards developing new efficient methods to neutralize their toxicity. The new proposed material proved its efficiency for the cyclophosphamide’s photo-oxidation. After 30 min of irradiation, 41.5% cyclophosphamide degraded under UV/silica microfiber decorated with TiO_2_. The Langmuir – Hinshelwood model accurately describes the photo-oxidation reaction. Also, the kinetic data of the photocatalytic process highlighted that the proposed material is a viable solution for advanced photo-oxidation of the chemotherapeutic drug, cyclophosphamide.

Thus, we proved that it is possible to give a superior usage to a valuable waste material – silica microfibers from optical fibers manufacturing industry. The new composite obtained offers an efficient and cheap method for neutralizing the effects of toxic pharmaceutical micropollutants from the wastewaters.

## Materials and Methods

All chemicals used, namely: methyl, ethyl, propyl, and t-butyl alcohol, methoxide, ethoxide, propoxide, and potassium t-butoxide, titanium (IV) methoxide, titanium (IV) ethoxide, titanium (IV) propoxide, tetraethyl orthotitanate, and tetrabutyl titanate were from Sigma Aldrich (Merck KGaA, Darmstadt, Germany). The silica microfibers were provided as waste from the processing of the optical glass fibers (Lanxess NV, Belgium). Ultrapure water was used (Millipore, Merck KGaA, Darmstadt, Germany).

For photocatalytic experiments N, N-bis(2-chloroethyl)-1,3,2-oxaza phosphinan-2-amine 2-oxide (cyclophosphamide-CP) (Sigma Aldrich – Merck KGaA, Darmstadt, Germany) and titanium dioxide (Degussa P25/Aeroxide TiO_2_ P25 – Merck KGaA, Darmstadt, Germany), trifluoroacetic anhydride, sodium chloride and toluene, all from Sigma Aldrich (Merck KGaA, Darmstadt, Germany) have been used.

A Sartorius installation (Sartorius Lab Instruments GmbH&Co, KG., Gottingen, Germany) facilitated the microfiltration of the mixtures obtained by the help of an ultrasonic bath (Elmasonic S10 H, Elma Schmidbauer GmbH, Singen, Germany). The structural investigations, SEM, and elemental analysis, EDX, have been performed on the FE-SEM Hitachi S4500 system (Hitachi High-Technologies Europe GmbH, Krefeld, Germany). The analysis performed on a Netzsch Thermal Analyzer (NETZSCH-Gerätebau GmbH, Selb, Germany) allowed the thermal stability characterization. The structural analysis by X rays diffraction, XRD, has been performed on an equipment Rigaku Ultima IV X-Ray Diffractometer (Rigaku Europe SE, Neu-Isenburg, Germany) using the Cu K_α_ radiation, λ = 1.5406 Å.

Taking into account the specific procedure proposed for nanotitania grafting onto silica microfibers, it is of interest to determine the dimensions of the crystalline nanoparticles. The average size (nm) of nano TiO_2_ crystallites (D) was estimated through the known Scherrer equation^56^ applied for spherical nanoparticle with a cubic lattice, according to Eq. (3):

3 $$D=\frac{0.94\,\lambda }{\beta \,\cos \,\theta }$$

where λ = 0.15406 nm is the X-ray wavelength of the incident radiation, β stands for the full width at half maximum expressed in radians ($=\beta (\deg .)\cdot \frac{\pi }{180}$), θ is the angle between the diffracted and incident beams (Bragg’s angle of diffraction), considering the Scherrer constant 0.94 that is specific for spherical particles with cubic symmetry^57^.

The ratio of rutile and anatase phase in the grafted nanotitania was evaluated by the help of Spurr-Meyers relationship^58^ as follows Eqs. (4) and (5):

4 $$ \% \,Content\,in\,anatase\,=\frac{100}{(1+1.26\frac{{I}_{R}}{{I}_{A}})}$$

5 $$ \% \,Content\,in\,rutile\,=\frac{100}{(1+0.80\frac{{I}_{A}}{{I}_{R}})}$$

where the considered maximum intensities I_A_ for anatase (corresponding to hkl = (101), where h, k, and l represent the Miller indices) and I_R_ for rutile phase (for hkl = (110)), corresponding to the diffraction angles of 12.63° for anatase maximum peak and 13.71° for rutile, respectively.

Zeta potentials and iso-electric point (IEP) values were determined using Zetasizer UltraPro (Malveren Pananalytical Instruments Ltd., Malveren, UK). It was used electrophoretic light scattering (ELS) for determining the ξ-potentials. For hydrodynamic particle size, it was applied the dynamic light scattering (DLS) technique.

The X-ray photoelectron spectroscopy (XPS) analysis performed in order to study the TiO_2_ grafting onto the silica microfibers surface provided data related to the interactions between the active nano oxide and the supporting silica oxide, and the surface oxygen species. The XPS measurements were run on PHOIBOS 150 ID-DLD system (SPECS Surface Nano Analysis GmbH, Germany) with a monochromatic aluminum anode X-ray source with the energy of 1486.71 eV, K α radiation, as the excitation source. The fixed analyzer transmission mode was applied. For peaks fitting, the SpecsLab Prodigy Version 4.43.2-r73078 (SPECS Surface Nano Analysis GmbH, Germany) was used.

In order to obtain information regarding the optical properties of the obtained SiO_2_ microfiber grafter with TiO_2,_ there were recorded the reflectance diffusion spectra UV-Vis (UV/DRS), which were obtained using a UV-Vis JASCO V-560 spectrometer (JASCO GmbH, Deutschland) equipped with a diffuse reflectance accessory. From the recorded DRS spectra could be calculated the energy of the band gap, thus assessing the capacity to absorb the UV radiation. The following equation gave the band gap energy Eq. (6):

6 $$E\frac{hc}{\lambda }$$

where *h* is Plank constant – 6.63 ⋅ 10^−34^ J ⋅ s, *c* is 3 ⋅ 10^8^ m ⋅ s^−1^, *λ* represents the minimum wavelength (cut-off value).

The analysis was run on the material obtained, and for comparison, it was performed the UV/DRS on silica microfiber and nanotitania Degussa P25 as reference.

The electronic characteristics of the synthesized material were analyzed through the Kubelka-Munk remission function^1^ as follows Eq. (7):

7 $$F(R)=\frac{K}{D}=\frac{{(1-R \% )}^{2}}{2\cdot R \% }$$

where K depends on the incident radiation energy, D represents the dispersion factor, R% is the diffused reflectance at a particular wavelength normalized to 100.

Then Eq. (8):

8 $$K=C{(E-{E}_{bg})}^{n}$$

where C stands for a material constant, E is the energy of the incident radiation, E_bg_ represents the band gap, and n takes different values based on the electronic transition type, namely n = 1/2 or 2 for permitted direct and indirect transition, respectively.

Thus Eq. (9):

9 $$F(R)=\frac{c{(E-{E}_{bg})}^{n}}{D}$$

and Eq. (10)

10 $$E\,=\,\frac{hc}{\lambda }\,=\,\frac{1236}{\lambda }\,(eV)$$

where *h* is Plank constant – 6.63 ⋅ 10^−34^ J ⋅ s, *c* is 3 ⋅ 10^8^ m ⋅ s^−1^.

Considering the situation for direct transition, the relationship (9) becomes Eq. (11):

11 $$F{(R)}^{2}\,=\,{(\frac{c}{D})}^{2}\,(E\,-\,{E}_{bg})$$

Representing the variation of ${(F(R)\cdot h\nu )}^{2}$ versus *E(eV)* for the direct electronic transition, it could be calculated the band gap value.

The BET surface areas of the synthesized material were determined by the help of N_2_ adsorption-desorption isotherms measured at 77 K using an ASAP 2020 Plus apparatus (Micromeritics Instruments, USA). N_2_ adsorption/desorption isotherms allowed the assessment of the specific surface area, pore size distributions, and isotherms for the prepared silica microfiber decorated with nano-titania. The Brunauer–Emmett–Teller (BET) procedure was applied to determine the specific surface area, using the adsorption data in the relative pressure (*P*/*P*0) range of 0.05–0.10. The desorption isotherms allowed the calculation of the pore size distribution.

The photocatalytic experiments were designed and run on a Heraeus type UV reactor equipped with a TQ-150-Z3 (λ = 320–550 nm) mercury lamp (from Heraeus Noblelight GmbH, Hanau, Germany). Agilent 7890 A gas chromatograph (Agilent Technologies Inc., Wilmington, USA) having a column with 5% diphenyl/95% dimethyl- polysiloxane stationary phase allowed to determine the CP’s concentration.

### *Procedure for preparing quartz fibers decorated with TiO*_2_

Figure S.11. from Supplementary Material introduces the applied method for preparing the silica fibers decorated with TiO_2_^59^. The experiments were developed using a three-necked flask (250 cm^3^) fitted with a reflux refrigerant. The experimental set-up was accommodated to a mechanical stirrer. 150 cm^3^ of the considered alcohol is added and then stirred with 0.054 moles of silica fibers powder until the transparency of the quartz microfibers occurs. At this particular moment, we introduced 0.054 moles of potassium alcoholate. The activation of the fibers’ surface is achieved during a half an hour refluxing process of the obtained mixture under continuous stir and heat. The heating stopped after the refluxing is over, but the stirring continued for another four hours. The reaction slurry was filtered on a Sartorius microfiltration system, and the excess of potassium alkoxide was removed by repeated washing with the alcohol considered. In parallel, into a 100 cm^3^ conical flask with a plunger stopper, 50 cm^3^ of the considered alcohol and 0.054 mol of titanium tetraalkoxide are ultrasonicated for one hour till the mixture is homogeneous.

As presented in Supplementary Material, Figure S.11, a quantity of 0.010 moles of activated silica microfiber is added, and the complex mixture homogenization is continued for three hours, to suspend the fibers and to complete the TiO_2_ gelling reaction onto their surface. A Sartorius microfiltration installation allowed the reaction mixture separation. The obtained silica microfiber decorated with titanium dioxide microparticles nanostructured was subsequently fully characterized.

### *Procedureforcyclophosphamidephoto-oxidation*

Photocatalytic experiments were performed using a Heraeus type UV reactor equipped with a TQ-150-Z3 (λ = 320–550 nm) mercury lamp. Before irradiation, samples were bubbled with air (50 L/h) in the dark for 30 minutes to avoid holes – electrons recombination.

The following reagents were used in photocatalytic experiments: cyclophosphamide (CP) (Sigma Aldrich), titanium dioxide (Degussa P25), and the synthesized silica microfibers decorated with titanium oxide nanostructured microparticles.

### *Analyticalmethods*

CP concentration was measured using an Agilent 7890 A gas chromatograph coupled with an Agilent 240 Ion Trap Mass Detector (GC-MS). As cyclophosphamide has low volatility, derivatization with trifluoroacetic anhydride was employed, forming N-tri fluoroacetyl- cyclophosphamide. Samples from 10 to 400 mL of aqueous samples were treated with sodium chloride (0.1 g NaCl/mL of the sample) at pH 10 buffer (0.5 mL buffer/mL of the sample) and then extracted with toluene. 1 mL extract was transferred in 2 mL GC vial and treated with 20 *μ*L of trifluoroacetic anhydride for derivatization. After sealing and vigorously shaking, vials were heated at 70 °C for 2 h. Subsequently to derivatization, 2 µL of toluene solution was analyzed by GC-MS. A column with 5% diphenyl/95% dimethyl- polysiloxane stationary phase (30 m, 0.25 mm ID, 0.25 μm film) was used for separation of trifluoroacetylated derivatives (RT: 9.88 min for IF derivative and RT:10.38 min for CF derivative). The experiments have been performed under specific working conditions for GC and mass detector, as follows: (a) GC conditions: splitless injection with injector temperature 250 °C, flow rate: 1 mL/min; carrier gas: helium 6.0; temperature ramp employed: 70 °C (1 min), 20 °C/min to 280, hold 2.5 min; (b) mass detector conditions: Ion Trap: 120 °C; Manifold: 50 °C; Transfer line: 250 °C; Ion source: 220 °C; Scan mode: single ion monitoring (quantified ion: m/z =  307 and qualifier ion: m/z=309).

### Ethical statement

This work does not contain studies with human participants or animals performed by any of the authors.

## Supplementary information

Supplementary Information.

## References

[1] Kim, E. J. Thorn-like TiO_2_ nanoarrays with broad spectrum antimicrobial activity through physical puncture and photocatalytic action. *Sci.Rep*. **9** (2019). 10.1038/s41598-019-50116-0 PMC6757029 31548584

[2] Dalod Antoine R M, Henriksen Lars, Grande Tor, Einarsrud Mari-Ann (2017). Functionalized TiO2 nanoparticles by single-step hydrothermal synthesis: the role of the silane coupling agents. Beilstein Journal of Nanotechnology.

[3] Anastasescu Crina, Preda Silviu, Rusu Adriana, Culita Dana, Plavan Gabriel, Strungaru Stefan, Calderon-Moreno Jose, Munteanu Cornel, Gifu Catalina, Enache Mirela, Socoteanu Radu, Angelescu Daniel, Anastasescu Mihai, Gartner Mariuca, Balint Ioan, Zaharescu Maria (2018). Tubular and Spherical SiO2 Obtained by Sol Gel Method for Lipase Immobilization and Enzymatic Activity. Molecules.

[4] Almasian Arash., Chizari Fard Ghazaleh., Maleknia Laleh. (2017). Fabrication of hollow and nonhollow SiO2nanofibers for removal of cationic dyes from aqueous solutions. Environmental Progress & Sustainable Energy.

[5] Polyakov Boris, Vlassov Sergei, Dorogin Leonid M, Butikova Jelena, Antsov Mikk, Oras Sven, Lõhmus Rünno, Kink Ilmar (2014). Manipulation of nanoparticles of different shapes inside a scanning electron microscope. Beilstein Journal of Nanotechnology.

[6] Schumann Erik, Hübner René, Grenzer Jörg, Gemming Sibylle, Krause Matthias (2018). Percolated Si:SiO2 Nanocomposites: Oven- vs. Millisecond Laser-Induced Crystallization of SiOx Thin Films. Nanomaterials.

[7] Nechifor AC, Totu EE, Ivan A, Danciulescu V, Sava S (2013). Microstructured adsorbent material: Silica dioxide - polyaniline for retaining aniline and chromium ions. Optoelectron. Adv. Mater. Rapid Commun..

[8] Sung Sang Do, Ojha Devi Prashad, You Ji Su, Lee Joori, Kim Jeongho, Lee Wan In (2015). 50 nm sized spherical TiO2nanocrystals for highly efficient mesoscopic perovskite solar cells. Nanoscale.

[9] Hosseini Arezoo, Kumar Pawan, Mahdi Najia, Zhang Yun, Shankar Karthik (2018). All-solid-state formation of titania nanotube arrays and their application in photoelectrochemical water splitting. Journal of Materials Science: Materials in Electronics.

[10] Yalavarthi Rambabu, Naldoni Alberto, Kment Štěpán, Mascaretti Luca, Kmentová Hana, Tomanec Ondřej, Schmuki Patrik, Zbořil Radek (2019). Radiative and Non-Radiative Recombination Pathways in Mixed-Phase TiO2 Nanotubes for PEC Water-Splitting. Catalysts.

[11] Caratão Bianca, Carneiro Edgar, Sá Pedro, Almeida Bernardo, Carvalho Sandra (2014). Properties of Electrospun TiO2Nanofibers. Journal of Nanotechnology.

[12] Seu-Run Kim SR, Imran Ali I, Kim JO (2019). Phenol degradation using an anodized graphene-doped TiO_2_ nanotube composite under visible light. Appl. Surf. Sci..

[13] Schneider Jenny, Matsuoka Masaya, Takeuchi Masato, Zhang Jinlong, Horiuchi Yu, Anpo Masakazu, Bahnemann Detlef W. (2014). Understanding TiO2Photocatalysis: Mechanisms and Materials. Chemical Reviews.

[14] Ohno Teruhisa, Akiyoshi Miyako, Umebayashi Tsutomu, Asai Keisuke, Mitsui Takahiro, Matsumura Michio (2004). Preparation of S-doped TiO2 photocatalysts and their photocatalytic activities under visible light. Applied Catalysis A: General.

[15] Shen Shaohua, Chen Jie, Wang Meng, Sheng Xia, Chen Xiangyan, Feng Xinjian, Mao Samuel S. (2018). Titanium dioxide nanostructures for photoelectrochemical applications. Progress in Materials Science.

[16] Fischer Kristina, Schulz Paulina, Atanasov Igor, Abdul Latif Amira, Thomas Isabell, Kühnert Mathias, Prager Andrea, Griebel Jan, Schulze Agnes (2018). Synthesis of High Crystalline TiO2 Nanoparticles on a Polymer Membrane to Degrade Pollutants from Water. Catalysts.

[17] Tavakolmoghadam Maryam, Mohammadi Toraj, Hemmati Mahmood, Naeimpour Fereshteh (2014). Surface modification of PVDF membranes by sputtered TiO2: fouling reduction potential in membrane bioreactors. Desalination and Water Treatment.

[18] Totu EE, Nechifor AC, Nechifor G, Aboul-Enein HY, Cristache CM (2017). Poly(methyl methacrylate) with TiO_2_ nanoparticles inclusion for stereolitographic complete denture manufacturing - the fututre in dental care for elderly edentulous patients?. J. Dent..

[19] Leandro, M. J., Edwards, J. C. W. & Cambridge, G. Clinical outcome in 22 patients with rheumatoid arthritistreated with B lymphocyte depletion. *Ann. Rheum. Dis*., 883–888 (2002). 10.1136/ard.61.10.883 PMC1753912 12228157

[20] Shah KM, Ohri AJ, A. U (2017). A Randomized Controlled Trial of Intravenous versus Oral Cyclophosphamide in Steroid-resistant Nephrotic Syndrome in Children. Indian J Nephrol..

[21] Mok CC (2016). Con: Cyclophosphamide for the treatment of lupus nephritis. Nephrol Dial Transplant..

[22] Ryerson CJ, Denton CP (2018). The fine line between success and failure in scleroderma lung fibrosis trials. Am. J. Respir. Crit. Care Med..

[23] Buerge Ignaz J., Buser Hans-Rudolf, Poiger Thomas, Müller Markus D. (2006). Occurrence and Fate of the Cytostatic Drugs Cyclophosphamide and Ifosfamide in Wastewater and Surface Waters†. Environmental Science & Technology.

[24] Webb Simon, Ternes Thomas, Gibert Michel, Olejniczak Klaus (2003). Indirect human exposure to pharmaceuticals via drinking water. Toxicology Letters.

[25] Sarder, A., Rabbani, M. G., Chowdhury, A. S. M. H. K. & Sobhani, M.-E. Molecular Basis of Drug Interactions of Methotrexate, Cyclophosphamide and 5-Fluorouracil as Chemotherapeutic Agents in Cancer. *Biomed. Res. Ther*., 10.7603/s40730-015-0005-1 (2016).

[26] Tsai-Turton M., Luong B. T., Tan Y., Luderer U. (2007). Cyclophosphamide-Induced Apoptosis in COV434 Human Granulosa Cells Involves Oxidative Stress and Glutathione Depletion. Toxicological Sciences.

[27] Sies Helmut (2017). Hydrogen peroxide as a central redox signaling molecule in physiological oxidative stress: Oxidative eustress. Redox Biology.

[28] Kot-Wasik A, Jakimska A, liwka-Kaszyńska M (2016). Occurrence and seasonal variations of 25 pharmaceutical residues in wastewater and drinking water treatment plants. Environ. Monit. Assess..

[29] Directive 2008/98/EC of the European Parliament and of the Council of 19 November 2008 on waste and Directive (EU) 2018/851 of the European Parliament and of the Council of 30 May 2018 amending Directive *2008/98/EC on waste*, http://data.europa.eu/eli/dir/ (2018).

[30] Lai WW-P, Chuang Y-C, Lin AY-C (2017). The effects and the toxicity increases caused by bicarbonate, chloride, and other water components during the UV/TiO_2_ degradation of oxazaphosphorine drugs. Environ. Sci. Pollut. Res..

[31] Franquet-Griell, H., Medina, A., Sans, C. & Lacorte, S. Biological and photochemical degradation of cytostatic drugs under laboratory conditions. *J. Hazard. Mater*. 319–328 (2017). 10.1016/j.jhazmat.2016.06.057 27421981

[32] Schulte-Oehlmann Ulrike, Oehlmann Jörg, Püttmann Wilhelm (2007). Humanpharmakawirkstoffe in der Umwelt: Einträge, Vorkommen und der Versuch einer Bestandsaufnahme. Umweltwissenschaften und Schadstoff-Forschung.

[33] Kümmerer Klaus, Al-Ahmad Ali (2009). Estimation of the cancer risk to humans resulting from the presence of cyclophosphamide and ifosfamide in surface water. Environmental Science and Pollution Research.

[34] Zhang Y, Xiao Y, Zhang J, Chang VWC, Lim T-T (2017). Degradation of cyclophosphamide and 5-fluorouracil in water using UV and UV/H_2_O_2_: Kinetics investigation, pathways and energetic analysis. J. Environ. Chem. Eng..

[35] Hromádko Luděk, Koudelková Eva, Bulánek Roman, Macak Jan M. (2017). SiO2 Fibers by Centrifugal Spinning with Excellent Textural Properties and Water Adsorption Performance. ACS Omega.

[36] *Growth opportunities in the European glass fiber*, www.researchandmarkets.com/reports/4663559/growth-opportunities-i. (2018).

[37] *E-glass Fiber Market Demand to Cross USD 12 Billion by 2025*, https://www.gminsights.com/industry-analysis/e-glass-fiber-market. (2019).

[38] Suttiponparnit K (2011). Role of Surface Area, Primary Particle Size, and Crystal Phase on Titanium Dioxide Nanoparticle Dispersion Properties. Nanoscale Res Lett..

[39] Li Q (2008). Antimicrobial nanomaterials for water disinfection and microbial control: potential applications and implications. Water Research..

[40] Low Jingxiang, Yu Jiaguo, Jaroniec Mietek, Wageh Swelm, Al-Ghamdi Ahmed A. (2017). Heterojunction Photocatalysts. Advanced Materials.

[41] Totu EE (2017). On physical and chemical characteristics of Poly(methylmethacrylate) nanocomposites for dental applications. I. Mater. Plast..

[42] Totu E, Segal E, Covington AK (1998). On the thermal behaviour of some polyimide membranes. J. Therm. Anal. Calorim..

[43] Hanaor Dorian A. H., Sorrell Charles C. (2013). Sand Supported Mixed-Phase TiO2Photocatalysts for Water Decontamination Applications. Advanced Engineering Materials.

[44] Xing Wang X (2018). Highly enhanced photocatalytic performance of TiO_2_ nanosheets through constructing TiO_2_/TiO_2_ quantum dots homojunction. Appl.Surf.Sci..

[45] Zhu Xiaodong, Han Shihu, Feng Wei, Kong Qingquan, Dong Zhihong, Wang Chenxi, Lei Jiahao, Yi Qian (2018). The effect of heat treatment on the anatase–rutile phase transformation and photocatalytic activity of Sn-doped TiO2 nanomaterials. RSC Advances.

[46] Krishnan P (2017). Characterization of photocatalytic TiO_2_ powder under varied environments using near ambient pressure X-ray photoelectron spectroscopy. Sci.Rep..

[47] Moulder, J. F., Stickle, W., Sobol, P. E. & Bomben, K. D. edited by J. C. *Handbook of X-ray Photoelectron Spectroscopy*. Perkin-Elmer, US (1992).

[48] Tan, R. *et al*. Theoretical study of the adsorption characteristics and the environmental influence of ornidazole on the surface of photocatalyst TiO_2_. *Sci.Rep*. **9**, (2019). 10.1038/s41598-019-47379-y PMC6659643 31350434

[49] Meng A, Zhang L, Cheng B, Yu J (2019). Photocatalysis. Adv. Mater..

[50] Liu Z, Zhang X, Murakami T, Fujishima A (2008). A Sol-gel SiO_2_/TiO_2_ bilayer films with self-cleaning and antireflection properties. Sol. Energy Mater. Sol. Cells..

[51] Sachs M (2019). Effect of oxygen deficiency on the excited state kinetics of WO3 and implications for photocatalysis. Chem. Sci..

[52] Nitoi Ines, Oancea Petruta, Cristea Ionut, Constsntin Lucian, Nechifor Gheorghe (2015). Kinetics and mechanism of chlorinated aniline degradation by TiO2 photocatalysis. Journal of Photochemistry and Photobiology A: Chemistry.

[53] Constantin LA, Cristea I, Nitoi I, Constantin AM, Nechifor G (2017). Kinetics of cyclophosphamide and ifosfamide degradation from aqueous system via TiO_2_ assisted photocatalysis. Rev. Chim..

[54] Constantin, L. A., Galaon, T., Chiriac, F. L., Constantin, M. A. & Cristea, N. I. UV-Vis Fe-TiO_2_ photo catalysis of cyclophosohamide and its degradation intermediates. *DBPapers. SGEM2017 Vienna GREEN Conference Proceedings*. 93–100, 10.5593/sgem2017H/63/S24.012 (2017).

[55] Lutterbeck CA, Machado EL, Kümmerer K (2015). Photodegradation of the antineoplastic cyclophosphamide: A comparative study of the efficiencies of UV/H_2_O_2_, UV/Fe_2_+/H_2_O_2_ and UV/TiO_2_ processes. Chemosphere.

[56] Pantazi A, Totu EE, Dorobantu D, Cristache CM, Enachescu M (2018). Poly(methyl metacrylate) Nanocomposites for Two-piece CAD/CAM Solution as an Alternative to Monolithic Removable Prosthesis. Mater. Plast..

[57] Langford J. I., Wilson A. J. C. (1978). Scherrer after sixty years: A survey and some new results in the determination of crystallite size. Journal of Applied Crystallography.

[58] Bessekhouad Yassine, Robert Didier, Weber Jean Victor (2003). Synthesis of photocatalytic TiO2 nanoparticles: optimization of the preparation conditions. Journal of Photochemistry and Photobiology A: Chemistry.

[59] Nechifor Gheorghe, Eftimie Totu Eugenia, Nechifor Aurelia Cristina, Isildak Ibrahim, Oprea Ovidiu, Cristache Corina Marilena (2019). Non-Resorbable Nanocomposite Membranes for Guided Bone Regeneration Based On Polysulfone-Quartz Fiber Grafted with Nano-TiO2. Nanomaterials.

