# Supplementary material for: Added value recyclability of glass fiber waste as photo-oxidation catalyst for toxic cytostatic micropollutants
Source: Sci Rep. 2020 Jan 10;10:136. doi: 10.1038/s41598-019-56836-7 (PMC6954219; doi:10.1038/s41598-019-56836-7)
Supplement: Supplementary file 1 — Supplementary Information. [file 41598_2019_56836_MOESM1_ESM.pdf]

SUPPLEMENTARY MATERIAL

Added value recyclability of glass fiber waste as photo-oxidation catalyst for toxic cytostatic micropollutants

Gheorghe Nechifor[1], Eugenia Eftimie Totu[1*], Aurelia Cristina Nechifor[1], Lucian Constantin[2], Alina Mirela Constantin[2], Mihaela Elena Cărăuşu[3], Ibrahim Isildak[4]

[1] Faculty of Applied Chemistry and Material Science, Polytechnic University of Bucharest, 060042 Bucharest, Romania
[2] National Research and Development Institute for Industrial Ecology – ECOIND Bucharest, 71-73 Drumul Podul Dambovitei Str., 060652, Bucharest, Romania
[3] Department of Public Health and Management, Faculty of Dental Medicine, Grigore T. Popa University of Medicine and Pharmacy, 700115 Iasi, Romania
[4] Department of Bioengineering, Faculty of Chemical and Metallurgical Engineering, Yildiz Technical University, 34210 Esenler-Istanbul, Turkey

*Correspondence: eugenia.totu@upb.ro

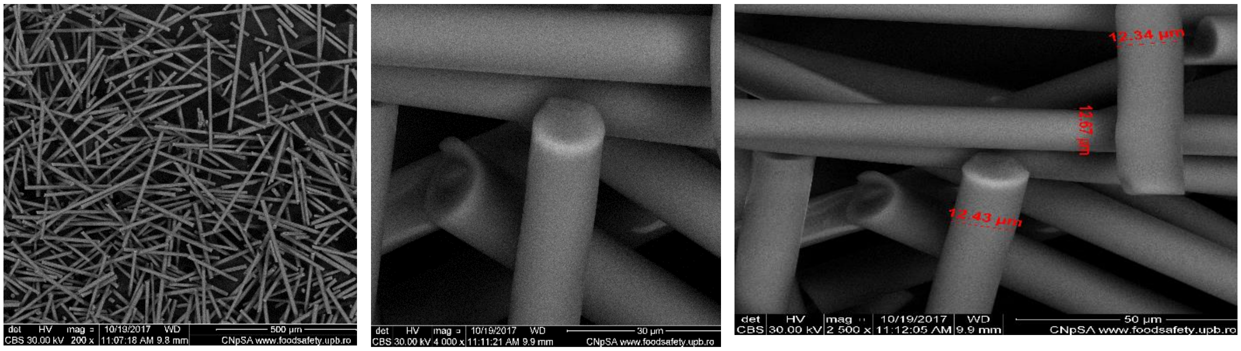

a. **b.** **c.**

Figure S.1. SEM images of silica microfibrils: a and b images at increasing resolutions; c. the dimensions of silica microfibers.

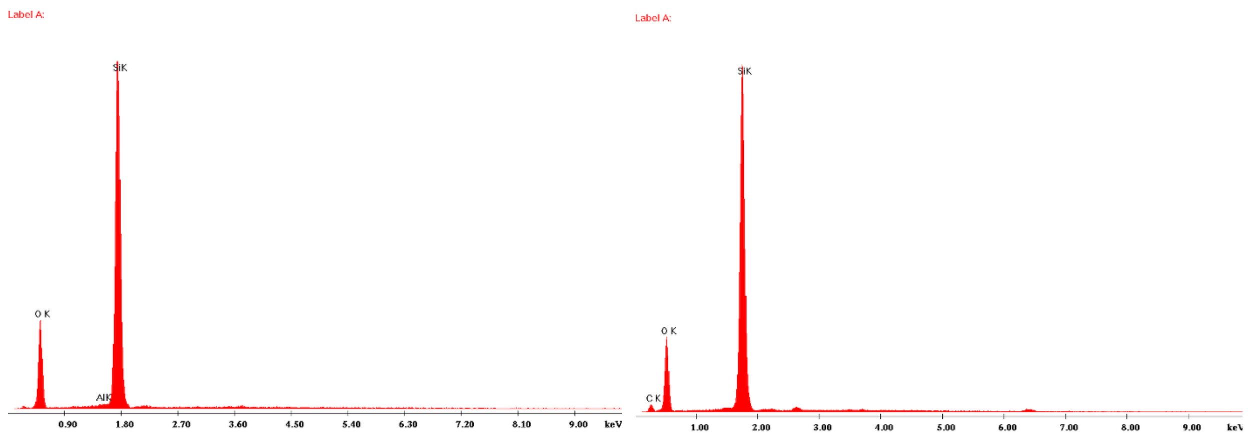

a. **b.**

Figure S.2. EDX Analysis of the silica microfibrils: before (a) and after activation (b) with potassium alkoxide in corresponding alcohol media.

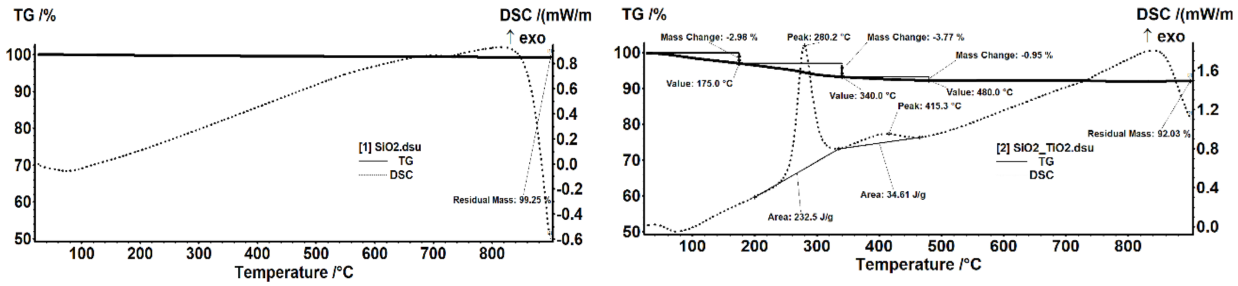

a **b**

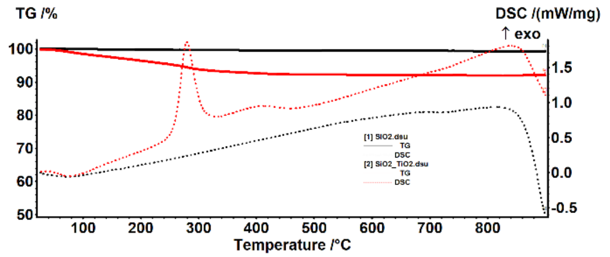

c

Figure S.3. Thermal analysis: (a) quartz microfibers; (b) quartz microfibers decorated with TiO_2; (c) overlapped thermograms of the quartz microfibers (black line) and quartz microfibers decorated with TiO_2 (red line) showing the different thermal behaviour.

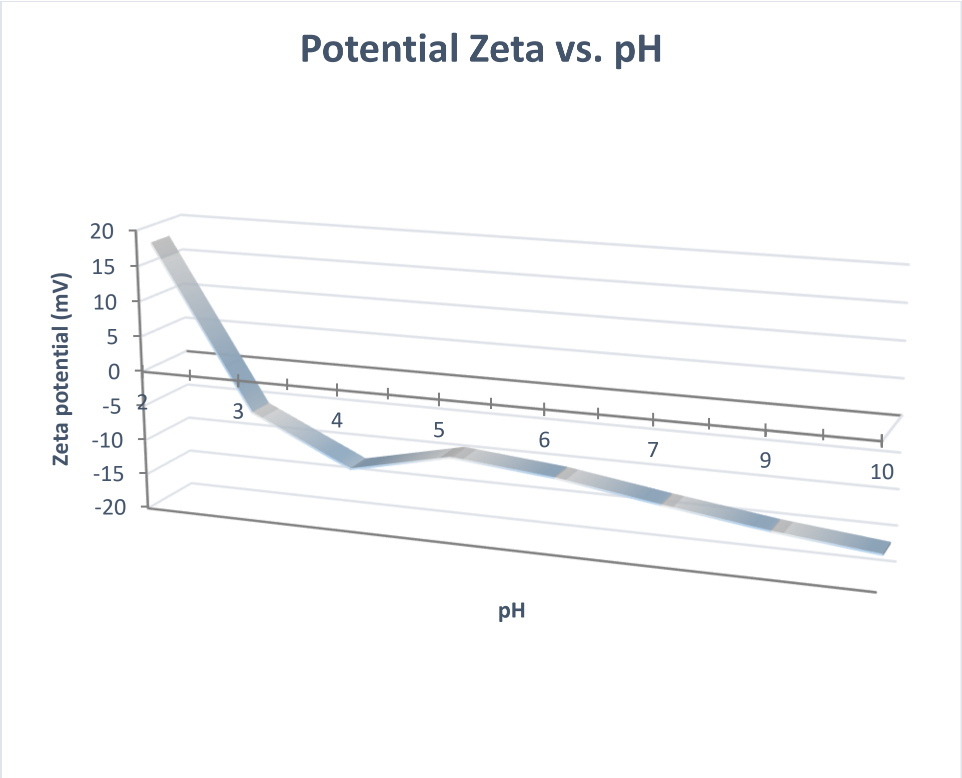

Figure S.4. Effect of pH variation on zeta potential for the surface of quartz fibers decorated with TiO_2.

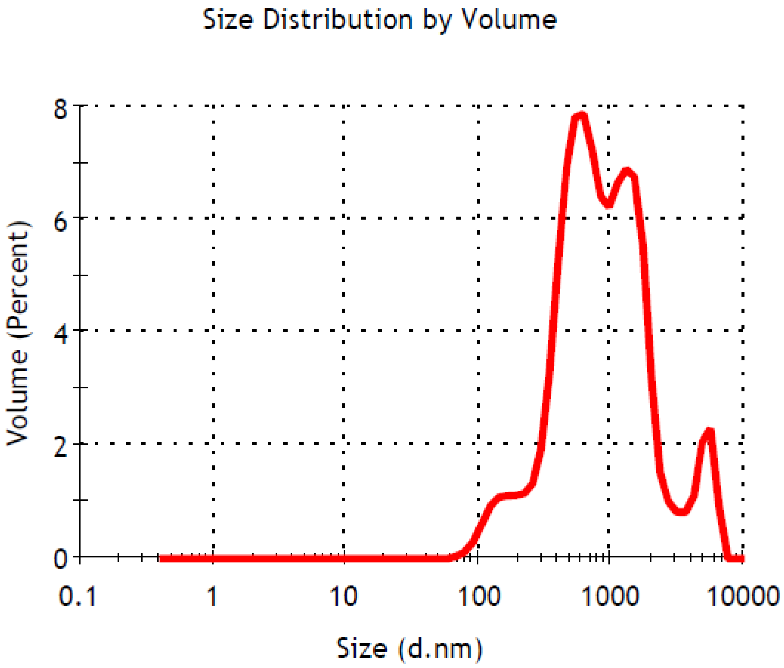

Figure S.5. Size distribution by volume for the surface of quartz fibers decorated with TiO_2.

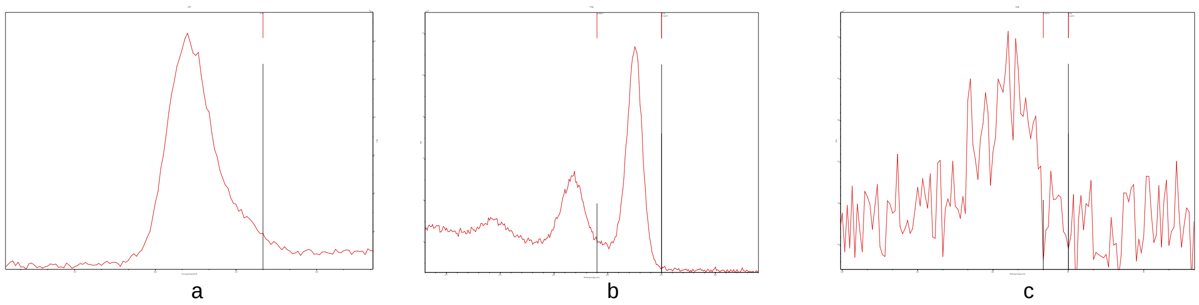

Figure S.6. XPS spectra of silica microfiber grafted with nanotitania, detailed for O1s (a), Ti2p (b) and Si 2p (c)

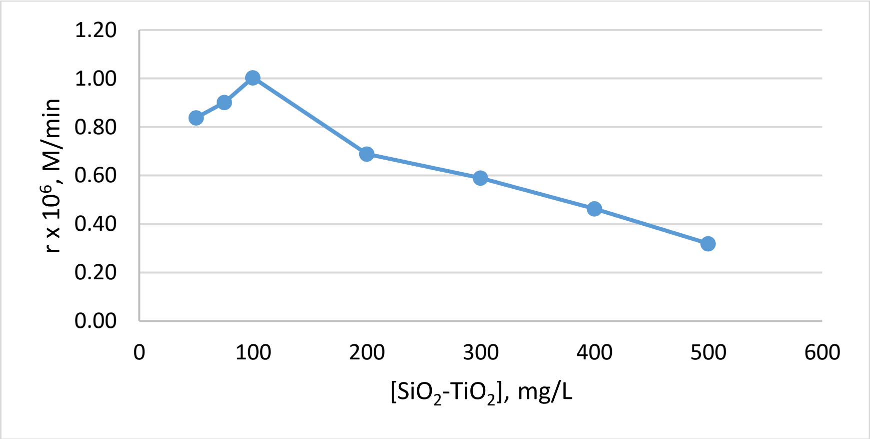

Figure S.7. Photochemical degradation reaction rate vs. catalyst amount

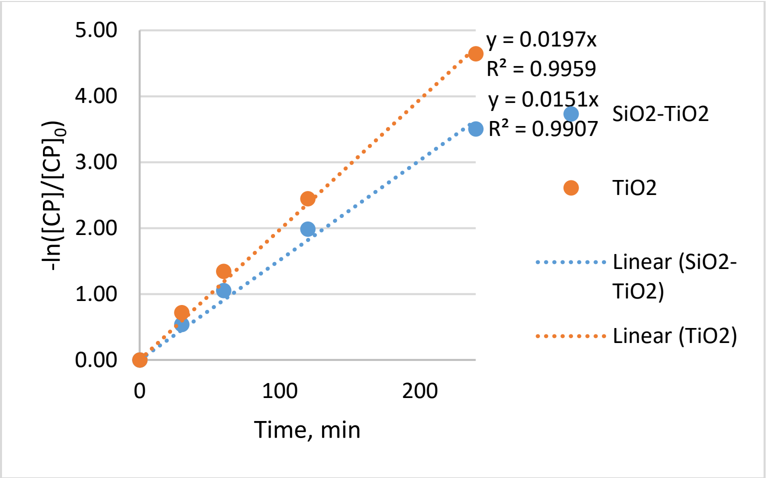

Figure S.8. Linearized first-order kinetic for CP photo-chemical degradation.

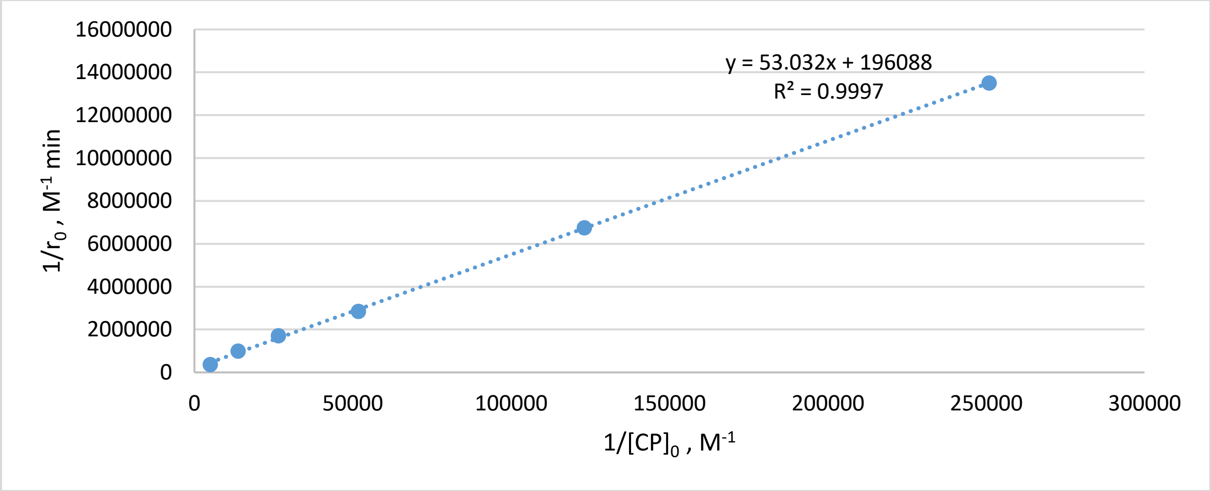

Figure S. 9. Linearization of Langmuir – Hinshelwood equation for CP photochemical degradation using SiO_2-TiO_2 catalyst

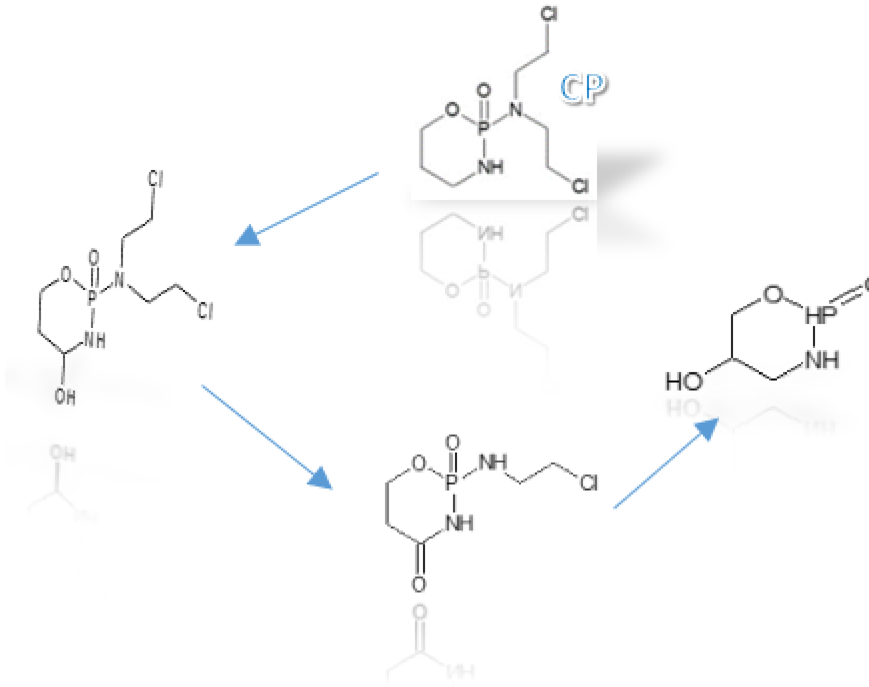

Figure S.10. Proposed assisted photodegradation pathway for cyclophosphamide under UV-Vis/Si microfibers decorated with nano-TiO_2 photocatalyst[1].

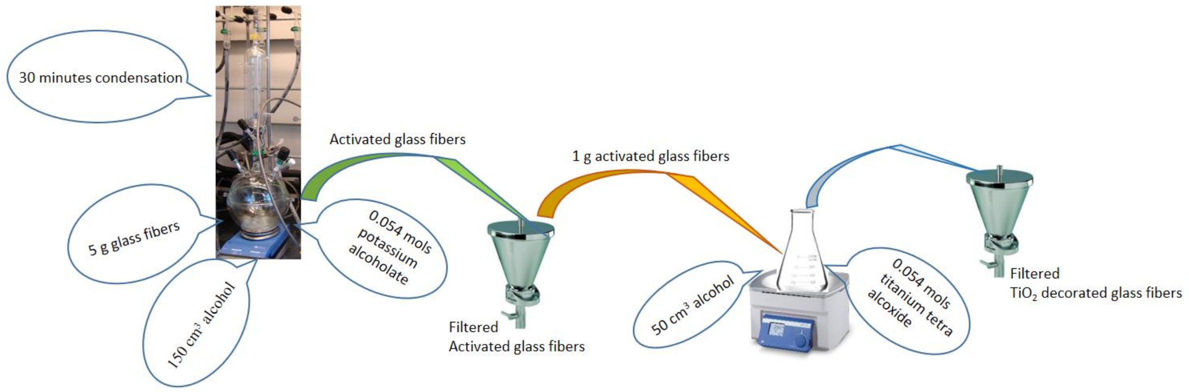

Figure S.11. Applied procedure for preparing quartz fibers decorated with TiO_2

The following scheme (Figure S.12.) presents synthetically the main aspects treated in the presented work, highlighting the recycle of silica waste into photocatalyst for photodegradation of toxic micropollutants as cyclophosphamide.

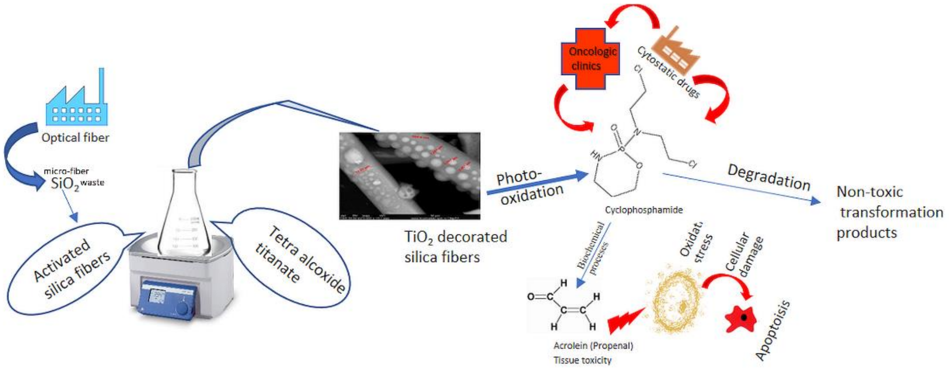

Figure S.12. Schematic representation of the presented work.

References

1. Constantin, L.A., Galaon, T., Chiriac, F.L., Constantin, M.A. and Cristea, N. I. UV-Vis Fe-TiO_2 photo catalysis of cyclophosphamide and its degradation intermediates. in *DBPapers. SGEM2017 Vienna GREEN Conference Proceedings.* 93–100 (2017). doi:10.5593/sgem2017H/63/S24.012
